# The reproducibility of heart Deformation Analysis for the evaluation of global cardiac function

**DOI:** 10.1186/1532-429X-18-S1-P17

**Published:** 2016-01-27

**Authors:** Eileen Hu-Wang, Haimanot Wasse, Jeremy Collins, James C Carr, Craig B Langman, Michael Markl, Kai Lin

**Affiliations:** 1 grid.465264.7Northwestern University Feinberg School of Medicine, Chicago, IL USA; 2 grid.465264.7Department of Nephrology, Northwestern University Feinberg School of Medicine, Chicago, IL USA; 3 grid.465264.7Department of Radiology, Northwestern University Feinberg School of Medicine, Chicago, IL USA

## Background

Analysis of global cardiac function is important for cardiovascular risk estimation but can be time-consuming. Using deformable image registration (DIR) algorithms, Heart Deformation Analysis (HDA) is a recently developed method for measuring cardiac function and motion on existing cardiac cine MR images. With a totally automatic workflow, HDA has the potential to be useful in clinical practice. In this study, the inter-study reproducibility of Heart Deformation Analysis will be analyzed.

## Methods

With IRB approval, 17 healthy volunteers were recruited to participate in the study. Each subject underwent two cardiac MR scans, the second scan taking place 14 days after the first scan. All participants underwent MRI examinations using the same cardiac cine protocol on a 1.5 T scanner (Avanto, Siemens AG, Germany). At four-chamber, two-chamber, and short-axis views, Segmented bSSFP cine sequences were acquired in the cardiac imaging planes with breath-holding. Imaging parameters were as follows: TR/TE = 2.8/1.1 ms; flip angle = 65^o^, voxel size = 2.1 × 2.1 × 8.0 mm^3^, bandwidth = 930 Hz/pixel. Eight to ten short-axis myocardial slices were set to cover the entire LV from base to apex. Each cine acquisition was acquired with retrospective ECG-gating and reconstructed into 25 phases. Left ventricular ejection fraction (LVEF) and left ventricular mass (LV mass) were automatically calculated by using the HDA tool. LVEF and LV mass were correlated between the two scans using SPSS software. SPSS intraclass correlation coefficient (ICC) was used to assess variations in LVEF and LV mass values between scans, with an alpha of 0.05 chosen to demarcate statistical significance.

## Results

**Figure 1:**
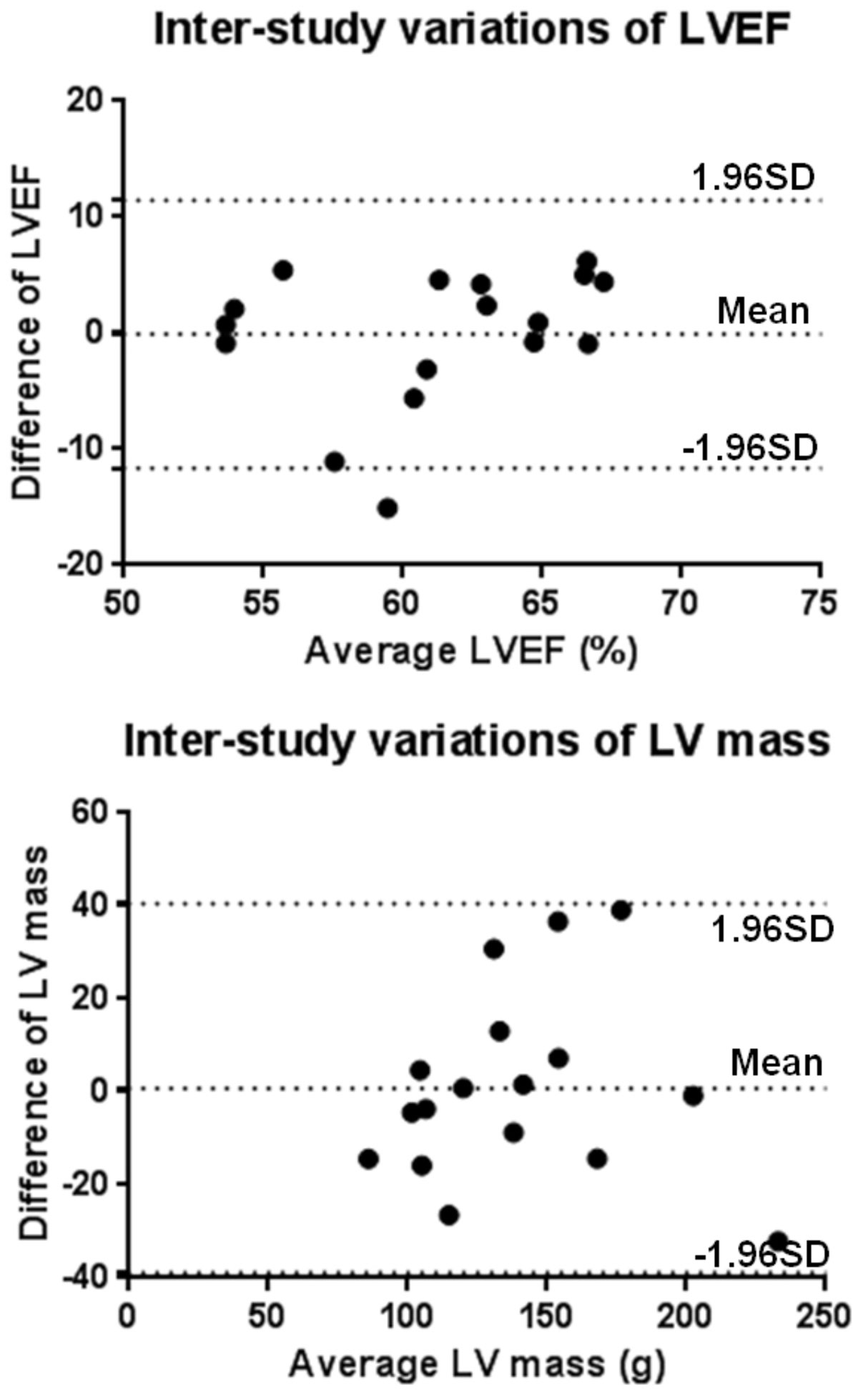
**Bland-Altman plots of inter-study variations of LVEF and LV mass measurements**.

A moderate to high correlation was found between LVEF and LV mass between the first and second scans. The LVEF ICC was found to be 0.636, p = 0.03. The LV mass ICC was found to be 0.527, p = 0.08. See figure 1 for the Bland-Altman plots showing inter-study variations of LVEF and LV mass measurements.

## Conclusions

Good inter-study reproducibility was found for evaluating global cardiac function. A moderate to high correlation was found between LVEF (ICC = 0.636) and LV mass (ICC = 0.527) between the first and second scans. There was statistical significance for correlation of LVEF between scans (p = 0.03) but no statistical significance was found for correlation of LV mass between the two scans (p = 0.08). The LV mass correlation was probably not found to be significant in this study because of the small sample size of 17 subjects; it is possible that a larger sample size could increase statistical power of the study.

In conclusion, the inter-study reproducibility and totally automatic workflow of HDA give this analysis method the potential to be useful in clinical practice, where a more time-efficient analysis method can be more easily incorporated into patient care.

